# Author Correction: Voltage-independent GluN2A-type NMDA receptor Ca^2+^ signaling promotes audiogenic seizures, attentional and cognitive deficits in mice

**DOI:** 10.1038/s42003-021-01717-x

**Published:** 2021-02-09

**Authors:** Ilaria Bertocchi, Ahmed Eltokhi, Andrey Rozov, Vivan Nguyễn Chi, Vidar Jensen, Thorsten Bus, Verena Pawlak, Marta Serafino, Hannah Sonntag, Boyi Yang, Nail Burnashev, Shi-Bin Li, Horst A. Obenhaus, Martin Both, Burkhard Niewoehner, Frank N. Single, Michael Briese, Thomas Boerner, Peter Gass, John Nick P. Rawlins, Georg Köhr, David M. Bannerman, Rolf Sprengel

**Affiliations:** 1 Departments Molecular Neurobiology and Physiology at the Max Planck Institute for Medical Research, Jahnstr. 29, 69120 Heidelberg, Germany; 2 Research Group of the Max Planck Institute for Medical Research at the Institute for Anatomy and Cell Biology of the Heidelberg University, Im Neuenheimer Feld 307, 69120 Heidelberg, Germany; 3 Department of Neuroscience Rita Levi Montalcini, University of Turin, Via Cherasco 15, 10126 Torino, Italy; 4 Neuroscience Institute–Cavalieri-Ottolenghi Foundation (NICO), Laboratory of Neuropsychopharmacology, Regionale Gonzole 10, 10043 Orbassano, Torino, Italy; 5 Department of Neurology and Epileptology, Hertie Institute for Clinical Brain Research, Eberhard Karls University Tübingen, Otfried-Müller Str. 27, 72076 Tübingen, Germany; 6 Department of Physiology and Pathophysiology, Heidelberg University, Im Neuenheimer Feld 326, 69120 Heidelberg, Germany; 7 OpenLab of Neurobiology, Kazan Federal University, 8 Kremlyovskaya Street, Kazan, 420008 Russian Federation; 8 Federal Center of Brain Research and Neurotechnologies, Ostrovityanova Str 1/10, Moscow, 117997 Russia; 9 Department of Molecular Medicine, Division of Physiology, Institute of Basic Medical Sciences, University of Oslo, Sognsvannsveien 9, 0372 Oslo, Norway; 10 Department of Behavior and Brain Organization, Research Center Caesar, Ludwig-Erhard-Allee 2, 53175 Bonn, Germany; 11 FARMA-DERMA s.r.l. Via dell’Artigiano 6-8, 40010 Sala Bolognese, Italy; 12 Department of Geriatrics, Tongji Hospital, Tongji Medical College, Huazhong University of Science and Technology, 1095 JieFang Road, Wuhan, Hubei 430030 China; 13 INSERM UMR 1249 Mediterranean Institute of Neurobiology (INMED), Aix-Marseille University, Parc Scientifique de Luminy, 163 avenue de Luminy BP13, 13273 Marseille Cedex 09, France; 14 Department of Psychiatry and Behavioral Sciences, Stanford University School of Medicine, 1201 Welch Road, Stanford, CA 94305 USA; 15 Wu Tsai Neurosciences Institute, Stanford University, Stanford Way, Rm E152, Stanford, CA 94305 USA; 16 Kavli Institute for Systems Neuroscience, Faculty of Medicine and Health Sciences, NTNU, Postboks 8905, NO-7491 Trondheim, Norway; 17 Department of Experimental Psychology, University of Oxford, Radcliffe Observatory, Anna Watts Building, Woodstock Rd, Oxford, OX2 6GG UK; 18 Miltenyi Biotec B.V. & Co. KG, Bergisch Gladbach, Friedrich-Ebert-Str. 68, 51429 Bergisch Gladbach, Germany; 19 Institute of Clinical Neurobiology, University Hospital Wuerzburg, Versbacherstraße 5, 97080 Wuerzburg, Germany; 20 RG Animal Models in Psychiatry, Animal Models Psychatry, Central Institute of Mental Health (CIMH), Faculty Mannheim, Heidelberg University, J5, 68159 Mannheim, Germany; 21 Department of Neurophysiology, Medical Faculty Mannheim, Heidelberg University, Ludolf-Krehl-Str. 13–17, 68167 Mannheim, Germany

**Keywords:** Channelopathies, Translational research

Correction to: *Communications Biology* 10.1038/s42003-020-01538-4, published online 8 January 2021.

The original version of this Article contained an error in the variant mentioned in the following text from the “Introduction”: “Notably, a similar Mg^2+^ block attenuating point mutation (c.1841A>G, p.Asn615Ser) at the identical position of the GluN2A subunit was found in two unrelated young female patients who suffered from epileptic seizures...”. The correct variant is (c.1845C>A, p.Asn615Lys).

The original version of this Article also contained an error in the mutation mentioned in the following text from the “Results” section “Generation of GluNA (N615S)-expressing mice”: “By classical gene-targeted replacement^28^ we inserted the c.1841A>G mutation at the homologous position in exon 10…”. The correct position of this mutation is c.1844A>G.

Both errors have been corrected in the HTML and PDF versions of the Article.

