# Supplementary material for: Voltage-independent GluN2A-type NMDA receptor Ca2+ signaling promotes audiogenic seizures, attentional and cognitive deficits in mice
Source: Commun Biol. 2021 Jan 8;4:59. doi: 10.1038/s42003-020-01538-4 (PMC7794508; doi:10.1038/s42003-020-01538-4)
Supplement: Supplementary file 2 — Supplementary Information [file 42003_2020_1538_MOESM2_ESM.pdf]

Supplementary Information

Voltage-independent GluN2A-type NMDA receptor Ca^{2+} signaling promotes audiogenic seizures, attentional and cognitive deficits in mice

Ilaria Bertocchi, Ahmed Eltokhi, Andrey Rozov, Vivan Nguyen Chi, Vidar Jensen, Thorsten Bus, Verena Pawlak, Marta Serafino, Hannah. Sonntag, Boyi Yang, Nail Burnashev, ShiBin Li, Horst A. Obenhaus, Martin Both, Burkhard Niewoehner, Frank N Single, Michael Briese, Thomas Boerner, Peter Gass, John Nick P. Rawlins, Georg Köhr, David M. Bannerman & Rolf Sprengel

Supplementary Information (available in this document)

Supplementary Figures 1 – 7
Supplementary Table 1
Supplementary Methods
Supplementary Statistics to main figures 2, 3, 7, 8, 9
Supplementary References

Supplementary Movies (linked for as downloads)

Video 1: **Clasping Reflex:**
Video 2: **Audiogenic seizures**
Video 3: **Hippocampal slice preparation**:

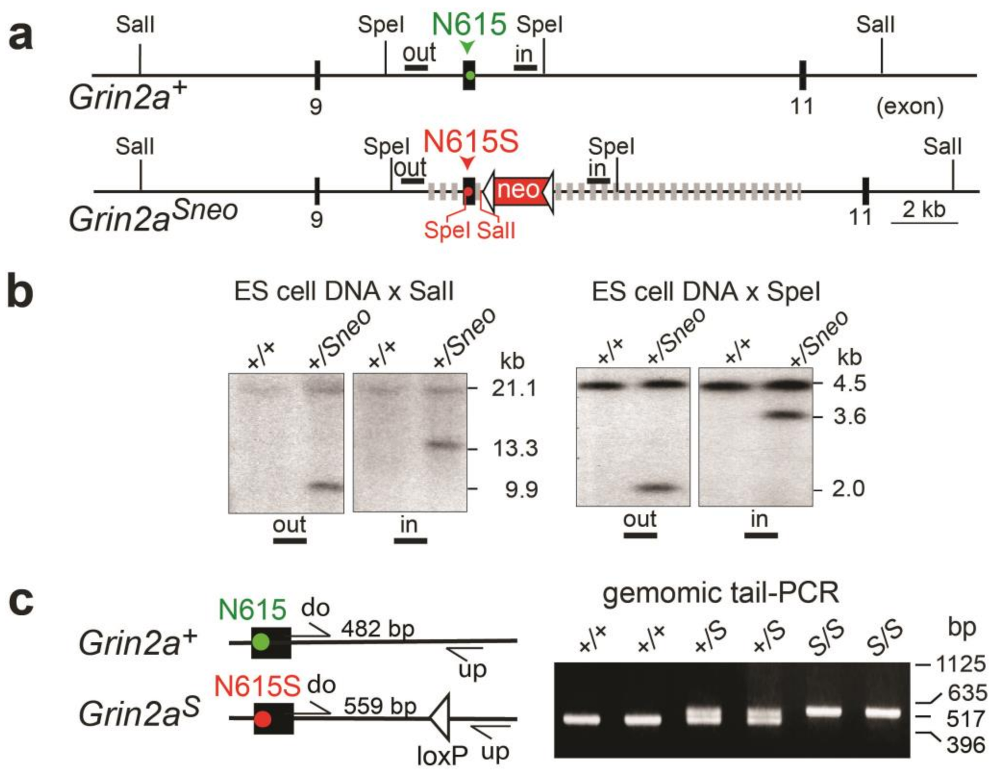

Supplementary Fig. 1: Gene-targeted knock-in of the GluN2A(N615S) point mutation.

a The gene segments encoding exon 9 to 11 of the *Grin2a* wild-type (*Grin2a*$^{+}$) and the mutated *Grin2a*^{Sneo} allele encoding the GluN2A(N615) subunit are depicted. The dashed region of *Grin2a*^{Sneo} allele gives the DNA segment isolated from a Sv129/J genomic library and that is covered by the gene-targeting vector. The floxed neomycin selection marker (neo) is still present in the targeted R1 ES-cells[1]. The positions of diagnostic restriction sites (SalI, SpeI) and southern blot probes (in) and (out) are indicated. **b** Southern blots of SalI- and SpeI-digested genomic DNA isolated from R1 ES cells (+/+) and the correctly-targeted ES cell clone *(+/Sneo*). The targeted ES cell clone was injected into B6D2F1/J blastocytes (Stock 100006; Jackson labs) to generate *Grin2a*$^{Sneo/+}$ mice. *Grin2a*$^{Sneo/+}$ were crossed subsequently with Cre-deleter mice (MGI: Tg(CMV-cre)1Cgn[2] for Cre-mediated deletion of the neo selection marker. Cre positive offspring in the first and second generation were analyzed by genomic tail-DNA PCR. **c** The primers 'do' (NR2A.NS1) and 'up' (2A-IN11UP1) amplify a 482 bp *Grin2a*$^{+}$ fragment and a 559 bp fragment, containing the remaining loxP site, from the *Grin2a*^{S} allele. The picture shows a run on a 3% w/v agarose gel of tail-PCR products of two animals per genotype.

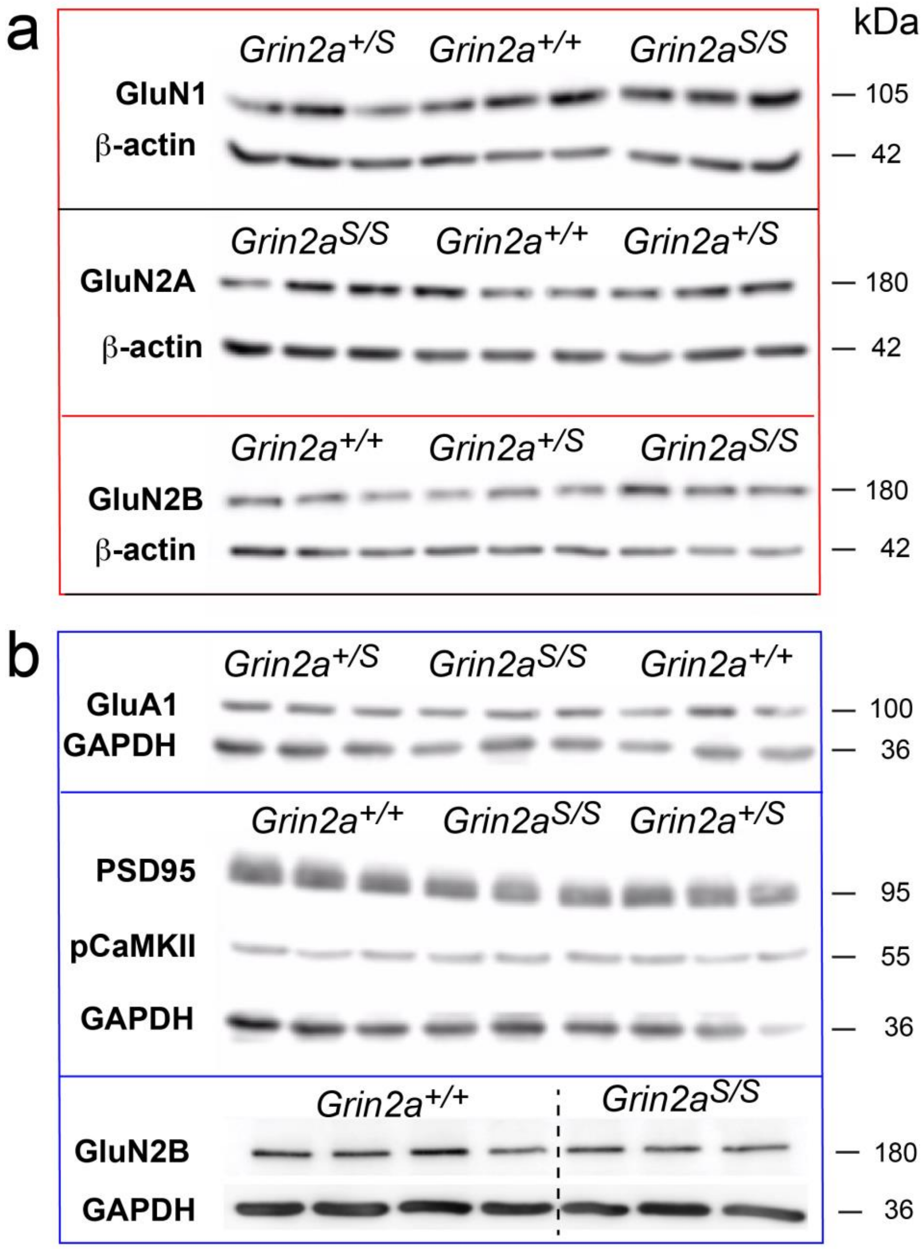

Supplementary Fig. 2: Expression levels of reference proteins in GluN2A(N615S) expressing mice

a Forebrain membrane fractions (10 μg) of from mice at P28 were analyzed in immunoblot for the presence of GluN1, Glun2A, GluN2B. The NMDAR subunits were visualized together with β-actin as a reference protein on blotted 8 % polyacrylamide gels. **b** Proteins levels of GluA1, PSD95, pCaMKII and GluN2b in total forebrain extract of 4 weeks old mice were determined in the immunoblots after PAGE in 8 or 12% polyacrylamide gels together with GAPDH as a reference. Antibodies used for protein detection are mentioned in the Method section of the main text.

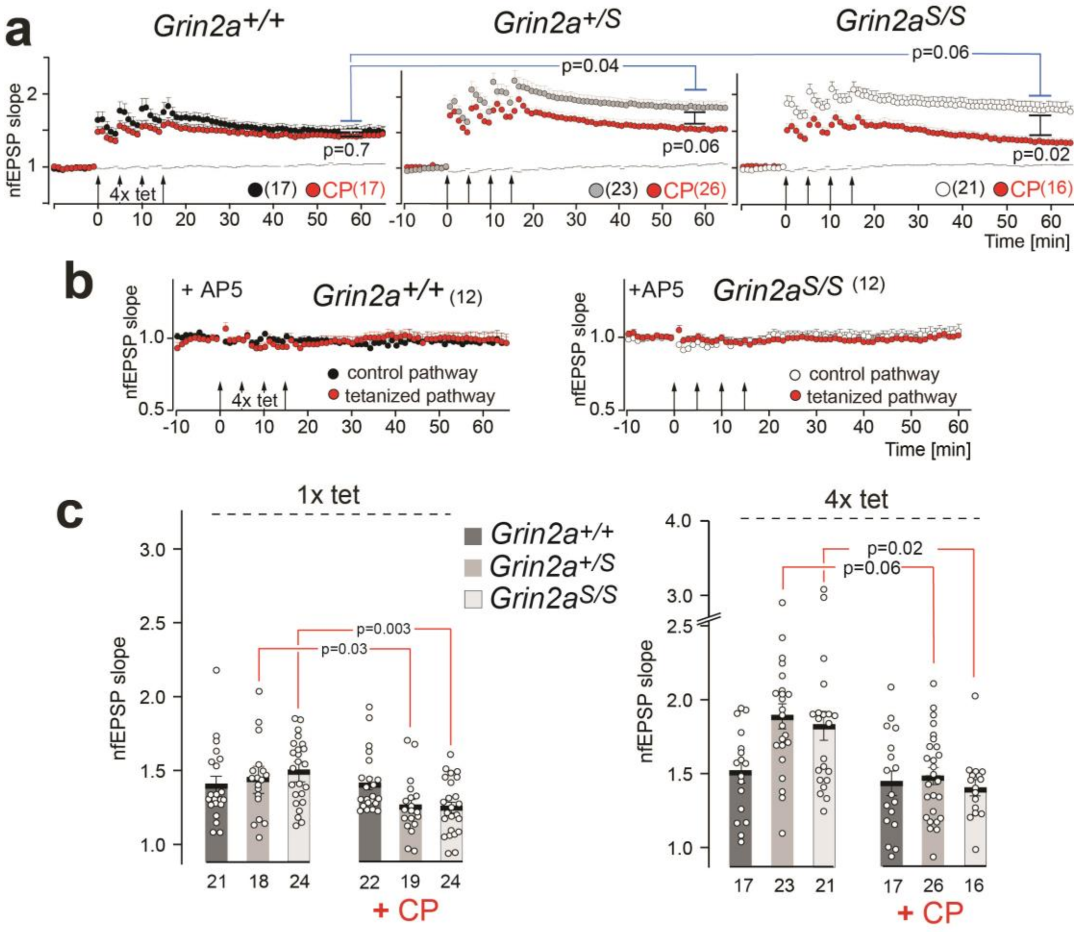

Supplementary Fig. 3: Field LTP is attenuated in GluN2A(N615) expressing mice in presence of the CP101,106.

a Field-LTP traces induced by 4x tet stimulation at CA3-to-CA1 synapsed in acute hippocampal slices were significantly enhanced in *Grin2a*$^{+/S}$ and *Grin2a*$^{S/S}$ mice compared to control littermates. In presence of the GluN2B specific antagonist CP101,606 (CP), the (4x tet)-fLTP was not affected in *Grin2a*$^{+/+}$ wild types but was significantly reduced in *Grin2a*$^{+/S}$ and *Grin2a*$^{S/S}$ littermates compared to the LTP traces of drug-naïve hippocampal slices. **b** Pharmacological inhibition of NMDAR signaling by AP5 abolished LTP in *Grin2a*$^{+/+}$ and *Grin2a*$^{S/S}$ mice. In all time courses, the normalized-fEPSP (nfEPSP) of the non-stimulated, control pathway is shown as dashed line. Black arrows indicate tetanic stimulations time points. **c** (left) fLTP values at CA3-to-CA1 synapses, as measured 40 – 45 min after tetanic stimulation (1 x 100 Hz; 1s), could be induced in all three genotypes and was specifically reduced by CP in mice expressing GluN2A(N615S3c *Grin2a*$^{+/+}$ (1.36 ± 0.06 vs. 1.00 ± 0.02); *Grin2a*$^{+/S}$ *(*1.40 ± 0.06 vs. 1.04 ± 0.02)*; Grin2a*$^{S/S}$ (1.45 ± 0.04 vs. 1.04 ± 0.02)*; Grin2a*$^{+/+}$ + CP(1.37 ± 0.04 vs. 1.02 ± 0.02)*; Grin2a*$^{+/S}$ + CP(1.21 ± 0.05 vs. 1.00 ± 0.02)*; Grin2a*$^{S/S}$ + CP (1.21 ± 0.04 vs. 0.95 ± 0.03). **c** (right) nfEPSP values of the 4x 100 Hz induced LTP in acute hippocampal slices: *Grin2a*$^{+/+}$ (1.48 ± 0.08 vs. 1.03 ± 0.03); *Grin2a*$^{+/S}$ (1.86 ± 0.09 vs. 1.05 ± 0.02); *Grin2a*$^{S/S}$ (1.79 ± 0.11 vs. 1.04 ± 0.02); *Grin2a*$^{+/}$, + CP (1.41 ± 0.09 vs. 1.04 ± 0.04); *Grin2a*$^{+/S}$ + CP (1.55 ± 0.08 vs. 0.97 ± 0.03; *Grin2a*$^{S/S}$, + CP (1.35 ± 0.06 vs. 0.97 ± 0.03); *Grin2a*$^{+/+}$ + AP5 (0.98 ± 0.05 vs. 0.96 ± 0.06) *Grin2a*$^{S/S}$ + AP5 (1.02 ± 0.05 vs. 1.01 ± 0.03). All data represent mean values ± SEM. For the comparison of genotypes / tetanization paradigms, 40 – 45 after tetanic stimulation, a linear mixed model test (SAS version 9.4) was used. The number of experiments is given in brackets in LTP traces. Numbers below bar graphs indicate number of brain slices. (For methods see main manuscript).

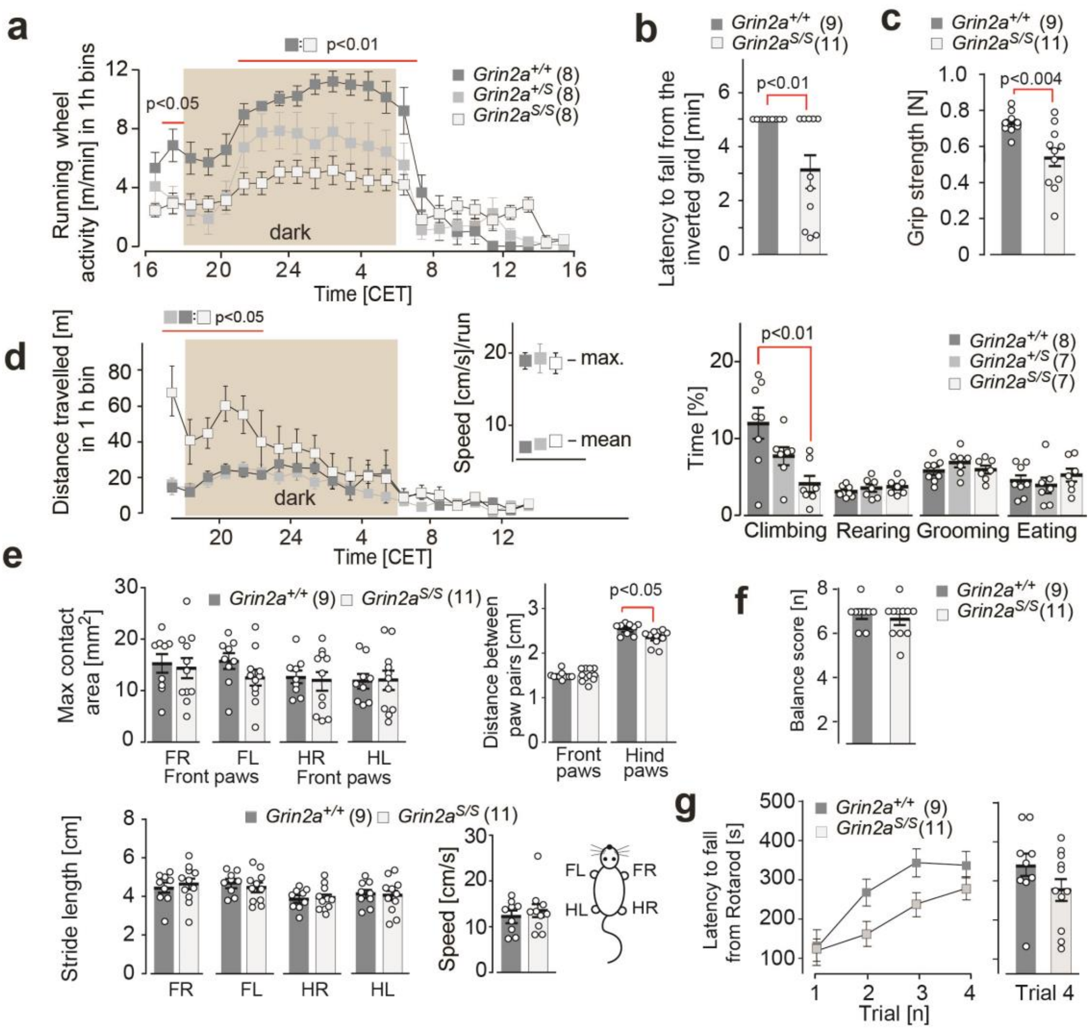

Supplementary Fig. 4: Reduced muscle strength and minor motor coordination impairments of *Grin2a*$^{S/S}$ mice.

a Lafayette wheel running activity during 24 h recordings in 1 h bins: *Grin2a*$^{S/S}$ vs. *Grin2a*$^{+/+}$. Two-way ANOVA main effect of genotype (F[2,21]=5.796, p=0.0099) and a strong effect of time (F[23,483]=48.9, p<0.0001) as well as a genotype by time interaction (F[46,483]=5.596, p<0.0001). The activity level of *Grin2a*$^{S/S}$ mice was significantly decreased at 5 p.m. (p<0.05) and from 9 p.m. to 6 a.m. (p<0.01, Bonferroni's post-tests). **b** In the inverted screen test the latency to fall was significantly reduced in *Grin2a*$^{S/S}$ mice (U=22.5, p<0.015). **c** The grip strength was lower *Grin2a*$^{S/S}$ vs. *Grin2a*$^{+/+}$ (F[1,18]=65535, p=0.008; F[1,18]=7.67, p=0.004, respectively). **d** However, in the first five hours in the unfamiliar LABORAS home cage was increase during 21h of the automated recording. The novelty-induced hyperactivity of *Grin2a*$^{S/S}$ mice could be recorded until 11 p.m. Two-way ANOVA revealed a main effect of genotype (F[2,19]=5.892, p=0.0102) with a strong effect of time (F[20,380]=15.01, p<0.0001) and a genotype by time interaction (F[40,380]=2.918, p<0.0001). Bonferroni's post-tests highlighted significant differences between 6 and 10 p.m. The mean and maximal speed (in mm/s) averaged for the entire 21-h session remained unaffected (mean: F[2,21]=3.127, p=0.067; max: F[2,21]=0.3359, p=0.719**)**. In the LABORAS the automated recording of climbing activity was significantly reduced in *Grin2a*$^{S/S}$ mice compared to heterozygous and WTs (F[2,21]=6.717, p=0.006; Bonferroni at p<0.01 for *Grin2a*$^{S/S}$ vs. control mice). Rearing, grooming and eating activity was similar in all three genotypes. **e** In the Catwalk test the *Grin2a*$^{S/S}$ mice showed comparable maximum contact areas and regular stride length of each individual footstep (p>0.05) when compared to WT. The average speed of runs was similar between both genotypes (p>0.05). The distance between the hind limps was narrower in *Grin2a*$^{S/S}$ mice indicating reduced base support (t[18]=2.85, p<0.01). FL, front left; FR, front right; HL, hind left; HR, hind right. **f** In the balance test *Grin2a*$^{S/S}$ mice scored successfully and similar compared to the control mice (U=41.5, p>0.5. **g.** The performance on the rotarod was not significantly different between the two genotypes after 4 days. Two-way ANOVA main effect between genotypes was (F[1,18]=3.790, p<0.0673 and the interaction between genotype and trials will be F[3,54]=1.808, p<0.1568). Number of mice is shown in brackets.

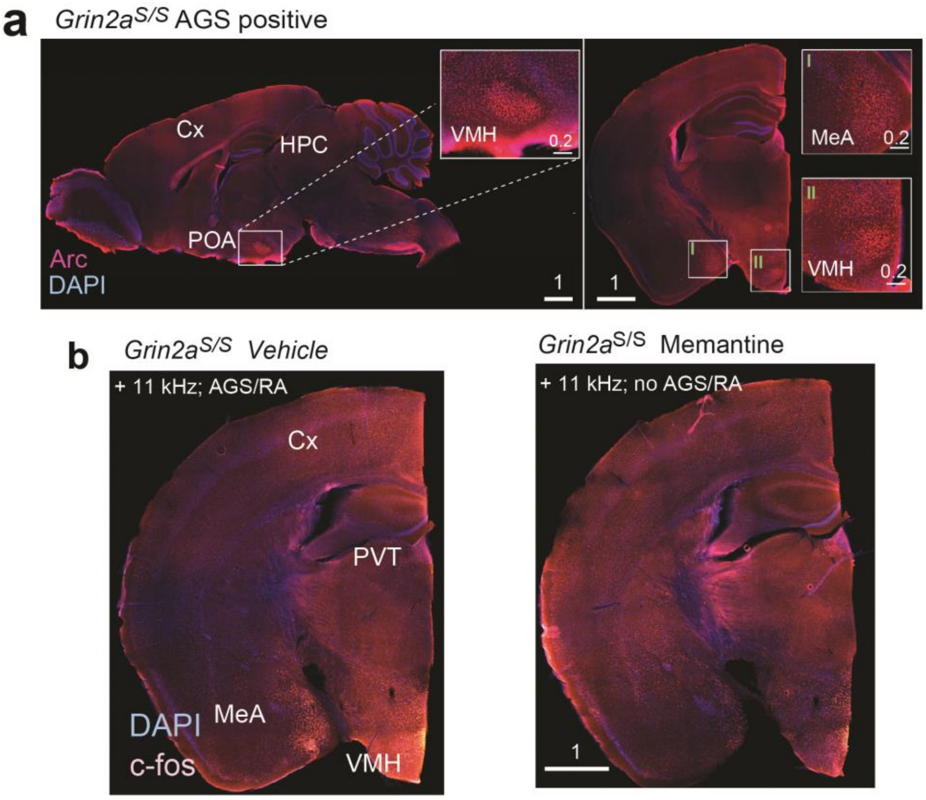

Supplementary Fig. 5: Audiogenic seizures (AGS) and memantine treatment in *Grin2a*$^{S/S}$ mice.

a ARC immunostaining in sagittal (left) and coronal brain sections (right) in AGS resuscitated *Grin2a*$^{S/S}$ mice. ARC expression was specifically enhanced in the ventromedial hypothalamus (VMH) and in the medial amygdala (MeA) (see also Fig. 4a,b). **b** I.p. vehicle injected mice showed AGS and enhanced c-Fos immunosignals in the MeA and VMH 120 min after tone exposure. The memantine injected, tone insensitive mice showed no enhanced c-Fos expression in the MeA and VMH (see also Fig. 4b,c). Scale bar = 1 mm.

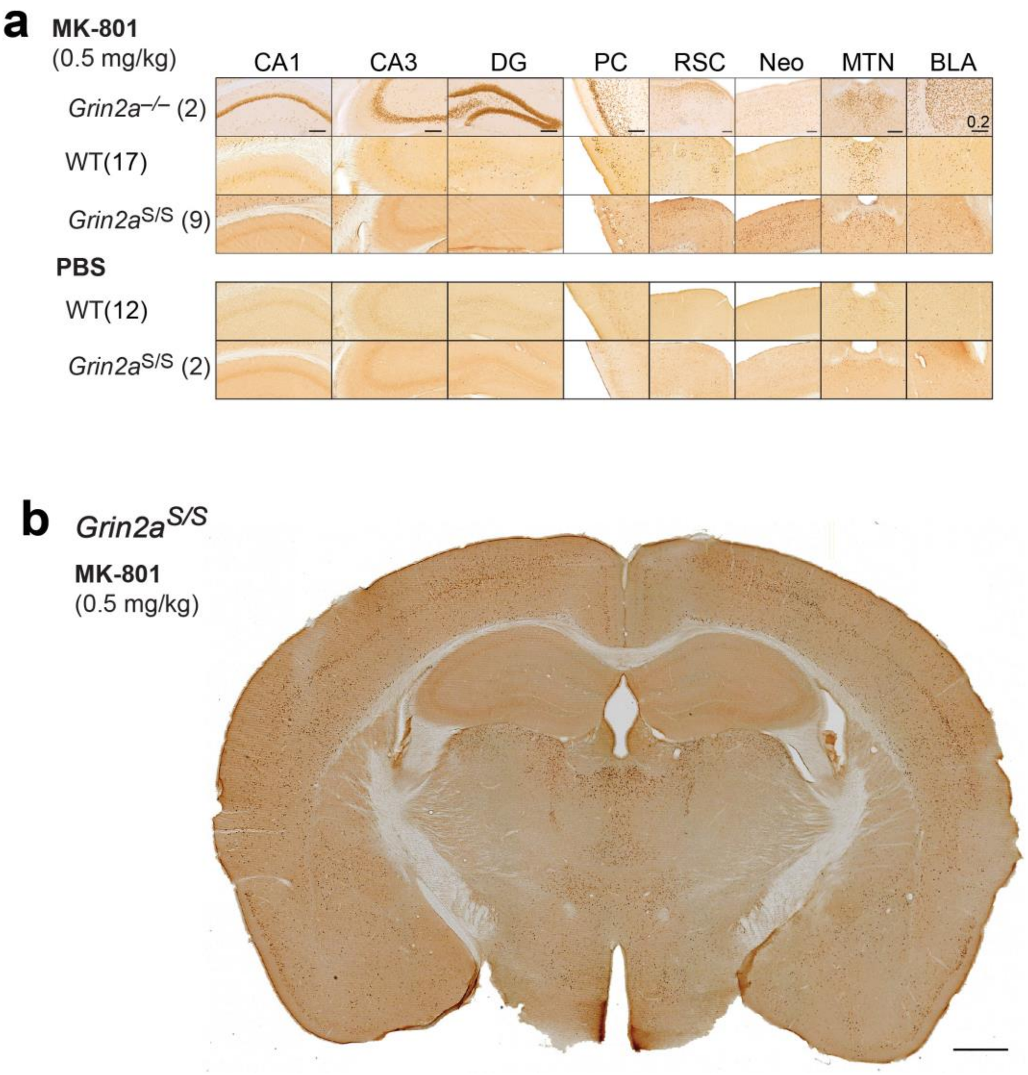

Supplementary Fig. 6: MK-801 induced c-Fos expression is reduced in DG granular and CA3 pyramidal cells of *Grin2a*$^{S/S}$ mice.

a C-Fos DAB immunosignals of MK-801 induced c-Fos expression in different genotypes and brain regions, as depicted in Fig. 5 in the main text. In vehicle (PBS) injected WT and *Grin2a*$^{S/S}$ mice c-Fos expression is weak and sparse. Similar weak c-Fos immunosignals were not included in the quantitative evaluation of the c-Fos expression shown in Fig. 5 in the main text. CA1 and CA3, *cornu ammonis* regions 1 and 3; DG, dentate gyrus; PC, piriform cortex; RSC: retrosplenial cortex; Neo, neocortex; MTN: midline thalamic nuclei; BLA, basolateral amygdala. Scale bar: 0.2 mm. **b** MK-801-induced c-Fos immunoreactivity in a coronal vibratome *Grin2a*$^{S/S}$ brain section; Scale bar: 0.5 mm.

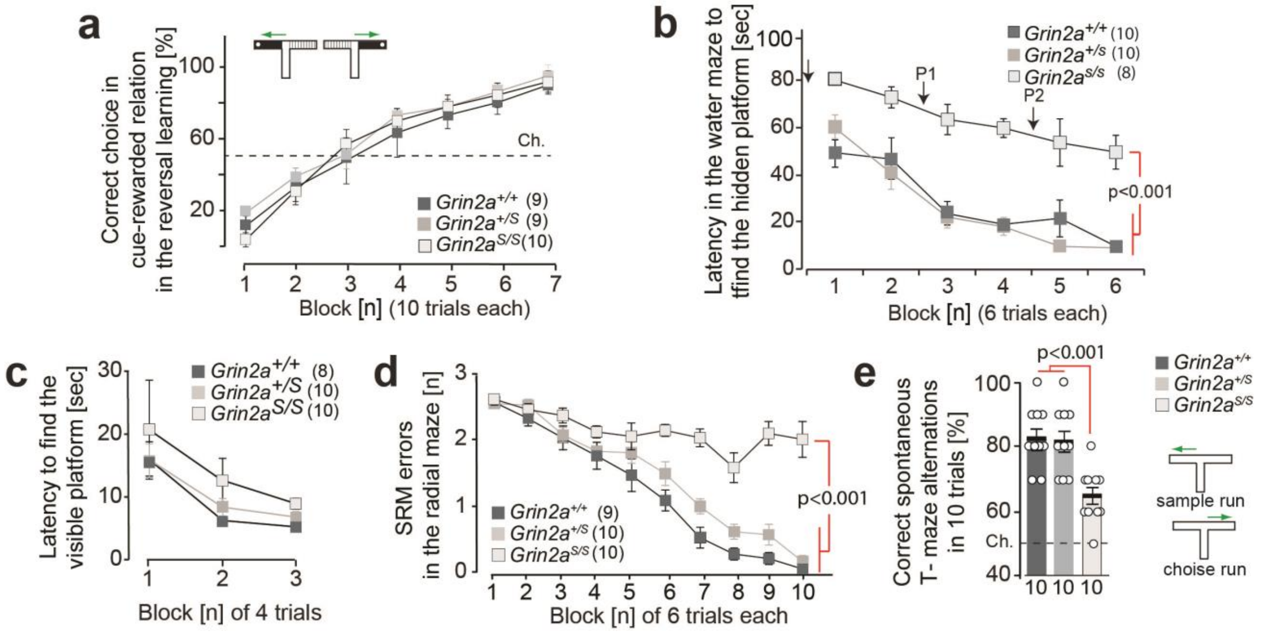

Supplementary Fig. 7: *Grin2a*$^{S/S}$ mice exhibit severe impairments in hippocampal cognition.

a In the T-maze visuo-tactile discrimination task, with marked goal arms, *Grin2a*$^{S/S}$ mutant mice learned to associate a specific visuo-tactile information (black foam versus light blue toweling) with a milk reward (see Fig. 8c). Now the mice had to learn in 70 trials that the previously unrewarded arm was now rewarded. In this task the relearning of the all genotypes were comparable. Analysis for the reversal phase: Main effect of genotype F(2,25) = 0.347; p = 0.71. Main effect of block F(7,175) = 105.826; p < 0.0001. Genotype by block F(14,175) = 0.528; p=0.9141. **b** The hidden version of the Morris water maze as presented in the main Fig. 8c, but now the latency instead of the path length the platform was analyzed. One-way ANOVA of the swim speed data revealed a significant difference between groups (F[2,27]=19.9; p<0.001) with the homozygous mutant mice swimming slower than the other two groups. *Post hoc* analysis showed a difference between homozygotes and both heterozygotes and WT (p<0.001). **c** In the visible version or the water maze all mice reached nearly identical levels of performance by the third block of testing, ANOVA of latencies for the visible platform task revealed a main effect of block (F(2,50)=17.9; p<0.001) but neither a main effect of group (F(2,25)=1.3; p>0.20), nor a groups by blocks interaction (F<1; p>0.20). **d** In the appetitively motivated six-arm radial maze both *Grin2a*$^{+/+}$ and *Grin2a*$^{+/S}$ mice learned the location of the food rewards but the *Grin2a*$^{S/S}$ mice showed little, if any, improvement. *Grin2a*$^{S/S}$ vs. *Grin2a*$^{+/+}$ mice; ANOVA revealed a significant main effect of group (F[2,26]=51.034; p<0.001), as well as a significant main effect of block (F[9,234]=84.820; p<0.001) and a significant group by block interaction (F[18,234]=9.852; p<0.001). Pairwise comparisons confirmed that the *Grin2a*$^{S/S}$ mice were significantly impaired relative to both WT and heterozygotes (both p<0.001), but also that there was a mild impairment in the heterozygotes compared to the controls (p<0.05). **e** In the T-maze task for spontaneous alternations *Grin2a*$^{S/S}$ mice were impaired *Grin2a*$^{S/S}$ vs. *Grin2a*$^{+/+}$ mice (main effect of genotype - spontaneous alternation F[2,27]=11.417; p<0.0003) pairwise Tukey's comparisons - p<0.01 for *Grin2a*$^{S/S}$ vs. both heterozygotes and WT. Number of mice is shown in brackets or within the bars of bar graphs.

	GluN1/2A	GluN1/2A(N615S)
$I_{(-60mv)}$/pA	1288 ± 285 (17)	1231 +265 (17)
Rise time/ms	3.3 ± 0.2 (10)	3.2 ± 0.1 (12)
τ des/ms *	152 ± 11 (5)	285 ±12 (10)
τ deacw	41 ± 3 (9)	34+5 (12)

Supplementary Table 1:
Electrophysiological signature of GluN1/2A and the mutant GluN1/2A(N615S) recombinant NMDARs in HEK293 cells. *The desensitization was significantly slower in the mutant compared to the wild-type NMDAR. I: Current ; pA: Picoamper; ms: milliseconds; τ des: desensitation, τ deacw: deactivation.

Supplementary Methods:

Genotyping
Mice were genotyped by tail-PCR with specific primers. Primers used were: do: 2A-TM3do (5'-GTG TGG GCC TTC TTT GCY GTC-3') and up: 2A-IN11UP1 (5'-CAT ATA TAC AAG CAT TGG AG-3'). Amplified gene fragments for the *Grin2a*^{S} and *Grin2a*$^{+}$ alleles are 559 and 482 bp, respectively.

LABORAS behavioral analysis
The activity of a single mouse in a novel home cage was analyzed for 24 h in the LABORAS cage (Metris). The LABORAS was calibrated using the calibration procedure and reference weights supplied by Metris. Each mouse was monitored and recorded for 24 h. In the LABORAS different behaviors of a mouse are analyzed by noninvasive recordings of the forces that are induced by the movements of the mouse[3].

Lafayette running wheel
For monitoring of voluntary running performance, the Lafayette running wheel (M86061, Lafayette instrument co.) was placed in a Macrolon type II home cage. The average speeds and total distance travelled during 24 h was automatically recorded using the activity wheel monitor (AWM) software (V 10.4; Lafayette instrument co.).

Grip strength measurement
The peak grip strength of forelimbs was measured using a grip-Strength Meter (Code47200, Ugo Basil). The mouse was placed over a base plate in front of a grasping triangle-shaped bar which was fitted to a force sensor connected to the peak amplifier. For each mouse the grip strength was determined three times and the mean value was calculated.

Gait analysis
The quantitative assessment of footfalls and motor performance in mice was measured using CatWalk^{Tm} software version XT 10.1 (Noldus Inc)[4-6]. Mouse had to cross an illuminated walkway with a glass floor (Noldus Inc) three times in a maximum of 12 s for the collection of the paw prints. For each mouse the N recording number was 3. The mean value was calculated for each mouse.

Inverted screen test

Muscle strength using all four limbs was tested in a modified version of the inverted screen test[7]. Here, the mouse was placed in the center of the lid of a Macrolon type II cage, rotated the lid to an inverted position and held it steadily 45 cm above an empty cage containing bedding. The latency to fall off the grid was recorded with a maximum trial time of 5 min.

Balance rod test
The static rods test[8] was used to evaluate the balance performance of the mice. The apparatus was composed of 7 rods with diameters of (6, 8, 12, 15, 20, 25 and 32 mm) and with a length of 50 cm and a height of 20 cm. The tested mouse was put on each rod for 10 s starting from the widest to the narrowest. The score was calculated by the number of rods from which the mouse tested did not fall off.

Rotarod performance test
Rotarod performance test was performed using a five lane rotarod treadmill (Code 47650; Ugo Basile)[8]. Mice were carefully placed on each lane of the rotarod and tested for two consecutive days, two trials per day with 1h time interval. Each trial lasted for 8 min while the rotarod was accelerated from 4 to 40 rpm. The latency to fall of each mouse was recorded.

Reversal of the simple cue discrimination task:
The reversal test was performed as the simple cue discrimination task was performed as described for the simple cue discrimination task in the main test. But now mice received 6 sessions comprising 10 trials per session with an ITI of approx. 15 – 20 min, and during session 7, the milk reward was placed into the food well only after the mouse had chosen the arm to ensure that the mice were unable to solve the task by smelling the reward.

Spontaneous alternation
The spontaneous alternations was analyzed as described before[9]. The apparatus consisted of an enclosed T-maze (each arm 30 x 10 x 29 cm), made of grey painted wood. A removable, central partition extended from the back wall of the T, 7 cm into the start arm, dividing the choice area and allowing access to only one goal arm at a time. Guillotine doors were present at the entrances to both goal arms. A thin layer of bedding was placed on the floor to facilitate running. In the sample phase of a trial, all the guillotine doors were raised, and the central partition was in place. A mouse was placed at the end of the start arm and allowed to make a free choice. The choice of goal arm was recorded. After choosing a goal arm, the mouse was confined there for 30 s. It was then immediately returned to the beginning of the start arm with the central partition now removed and all guillotine doors raised. The mouse was again allowed to make a free choice of either goal arm (choice phase). If the mouse entered the opposite goal arm to that visited during the first phase, this was scored as an alternation. Each mouse received 10 trials over 3 days; the minimum inter trial interval was approximately 5 min.

Morris Water
(see main text)

Visible platform water maze
Mice were trained to find a visible escape platform, which had a variable spatial location, as described previously[10]. Here, the escape platform was clearly recognizable for the mouse. The platform was now located 1 cm above the water surface and was signaled by the presence of a black and white striped cylinder. Both the platform position and the start location (always opposite to the escape platform) were varied randomly from trial to trial. Mice received 6 trials per day for 3 days, with an inter-trial interval (ITI) of approximately 15 sec. Mice were allowed to find the visible escape platform within a maximum of 90 sec. Any mouse that failed to find the platform within the allotted time was lifted out of the water by the experimenter and placed onto the platform for 30 sec. Path lengths (m) and latencies (sec) to escape from water onto the visible platform were analyzed.

Spatial reference memory (SRM)
Hippocampus-dependent SRM was assessed also in the elevated radial maze as we have described in detail[11] and by using the identical equipment. In the radial maze, three of the six arms were baited with a 0.1 ml sweetened condensed milk. The same 3 out of 6 arms (defined by the allocentric spatial cues)

were always baited for a given mouse and were allocated such that two of these arms were adjacent and the third was between two non-rewarded arms (e.g. arms 1, 2 and 4). Different combinations of arms were used as far as possible, although the arm allocations were counterbalanced across groups. Mice were placed on the central platform and allowed to explore the maze until they had collected the three milk rewards. They were then removed from the maze and returned to their home cages between trials. Perspex doors prevented mice from re-entering an arm that they had already visited on that trial. All the doors were closed each time the mouse returned to the central platform, and confined the mouse there for 5 s until the next choice. Once an arm had been visited, its door remained closed for subsequent choices. Thus, all 6 doors were open for the first choice, 5 for the second choice, 4 for the third choice, and so on. Using this testing procedure it was not possible for the mice to make working memory errors. Reference memory errors were defined as entries into arms that were never baited (maximum of 3 errors per trial). Entry into an arm was defined when a mouse placed all 4 paws into that arm. The maze was rotated periodically to prevent the mice from using intramaze cues to solve the task. Mice received 60 trials in total (6 trials/day where possible). Data were arranged in 10 blocks of 6 trials for analysis.

Supplementary Statistics to Fig. 1, 2, 3, 4, 7 and 8

All data are given as ±SEM.

Fig. 1I GluN2A(N615S) containing NMDAR expression *in vitro* and *in vivo*

e For the quantification of the proteins the area of the positive immunosignals (see Supplementary Fig.2) were quantified using Image J and their relative expression to either beta-actin or GAPDH of each proteins was statistically evaluated by one-way ANOVA followed by **Turkey's** multiple comparison test. P-values are given for the *Grin2a*$^{+/+}$ vs. *Grin2a*$^{+/S}$ and (&) for *Grin2a*$^{+/+}$ vs. *Grin2a*$^{S/S}$. In the membrane fraction: (GluN1: 0.06 & 0.45; GluN2A: 0.88 & 0.73; GluN2B: 0.84 & 0.0005); (GluN2B: *Grin2a*$^{+S}$ vs. *Grin2a*$^{S/S}$: 0.0007). For total proteins: (GluA1: 0.19 & 0.70; PSD95: 0.34 & 0.96; pCaMKII: 0.33 & 0.96; GluN2B: Unpaired t-Test *Grin2a*$^{+/+}$ vs. *Grin2a*$^{S/S}$: 0.56)

Fig. 2: Hippocampal synaptic transmission and plasticity in *Grin2a*$^{S/S}$ and *Grin2a*$^{+/S}$ mice with GluN2A(N615S) mutation

b Data of the same experimental group were pooled across animals and are presented as mean ± SEM (see also[12]). Statistical significance was evaluated by using a linear mixed model analysis (SAS 9.1) with p<0.05 being designated as statistically significant.

c, e Data represent mean values ± SEM. For the comparison of genotypes / tetanization paradigms, 40 – 45 after tetanic stimulation, a linear mixed model test (SAS version 9.4) was used. For detailed statistics see Supplementary Fig. 3c.

d Filed fLTP traces from in freely moving mice were comparable between *Grin2a*$^{S/S}$ and *Grin2a*$^{+/+}$ mice as indicated by repeated measures ANOVA, comparing fEPSPs from 60 – 180 min after LTP induction (p=0.118). Field-EPSPs were significantly enhanced 180 min after LTP induction compared to baseline in WT (p=0.017) and in the *Grin2a*$^{S/S}$ mice (p=0.030).

Fig. 3: *Grin2a*$^{S/S}$ mice are viable and show no signs of neurodegeneration or altered brain structure.

a The body weight of *Grin2a*$^{S/S}$ was about 18 % reduced (One-way ANOVA, F[2,67]=11.74, p<0.0001, Bonferroni comparison at p<0.01 for het vs. hom and at p<0.001 for wt vs. hom). Reduced nesting *Grin2a*$^{S/S}$ vs. *Grin2a*$^{+/+}$ littermates (one-way ANOVA followed by Tukey's multiple comparison test F[2,40]=16,69, P<0.001). Reduced burrowing *Grin2a*$^{S/S}$ vs. *Grin2a*$^{+/+}$ (two-way RM ANOVA followed by Bonferroni post-test, significant main effect of genotype X time interaction F[2,62]=9,67, P<0.001).

c Lafayette distance travelled: *Grin2a*$^{S/S}$ vs. *Grin2a*$^{+/+}$; (one-way ANOVA: F[2,23]=7.87, p=0.0028, Bonferroni's test at p<0.01.

Fig. 4: *Grin2a*$^{S/S}$ mice are susceptible to brainstem-derived AGS that can be rescued by NMDAR antagonists

a The Log-rank (Mantel-Cox) test indicated the difference in the mortality curve as p<0.0001 at the end of AGS induction protocol and **c,** as p<0.002 50 h after memantine injection.

e The Log-rank (Mantel-Cox) test indicated the difference in the mortality curves as p = 0.0404, measured 100 h after MK801 injection.

Fig. 7: *Grin2a*$^{S/S}$ mice are hyperactive and show increased attention and lack of inhibition

a *Grin2a*$^{S/S}$ displayed a higher degree of spontaneous locomotor activity compared to wild-types and *Grin2a*$^{+/S}$, indicating that *Grin2a*$^{S/S}$ mice are hyperactive (*Grin2a*$^{+/+}$ vs. *Grin2a*$^{+/S}$ vs. *Grin2a*$^{S/S}$: 1606.9 ± 256.023: 1038.1 ± 179.234: 6268.50 ± 901.031 Mean ± SEM). A Kruskal-Wallis ANOVA revealed a significant main effect of group (H(2)=18.715; p<0.001). Further analysis using Dunn's method of multiple pairwise comparisons showed that this was due to significant differences between *Grin2a*$^{+/+}$ and *Grin2a*$^{S/S}$ (p<.0.05) as well as *Grin2a*$^{S/S}$ and *Grin2a*$^{+/S}$ (p<0.05), though there was no significant difference between *Grin2a*$^{+/+}$ and *Grin2a*$^{+/S}$ (p>0.05).

b Novel environment induced activity: *Grin2a*$^{S/S}$ vs. *Grin2a*$^{+/+}$; (Kruskal Wallis test; H(2) = 23.084; p<0.001; Dunn's post-hoc comparisons, p<0.05 for *Grin2a*$^{S/S}$ mice vs. both other groups). **b** Decreased habituation in three exposure sessions to 5 novel objects: *Grin2a*$^{S/S}$ vs. *Grin2a*$^{+/+}$ + *Grin2a*$^{+/S}$: main effect of genotype: F[2,16]=31.86, p<0.0001; not a main effect of session: F[3,48]=1.1729, p=0.1736; genotype x session interaction: F[6,48]=7.657, p<0.0001. Bonferroni's post-tests: *Grin2a*$^{+/+}$ vs. *Grin2a*$^{+/S}$: n.s.; *Grin2a*$^{+/S}$ vs. *Grin2a*$^{S/S}$: Session 2-4: p<0.001; *Grin2a*$^{+/+}$ vs. *Grin2a*$^{S/S}$ – Session 2: p<0.01 / Session 2+3: p<0.001.

c Reduced latency first fall in CAR: *Grin2a*$^{S/S}$ and *Grin2a*$^{+/+}$ (n=11-17; Log-rank (Mantel-Cox) Test, and significantly more falls from the platform (One-way ANOVA followed by Tukey's multiple comparison test F[2,40]= 11,28, P<0.001).

d Decrease social Interaction. *Grin2a*$^{S/S}$ vs. *Grin2a*$^{+/+}$ (One-way ANOVA followed by Tukey's multiple comparison test F[2,38]= 7,73, P<0.01).

e Novel object recognition test. *Grin2a*$^{S/S}$ mice made significantly more interactions with the two objects in both the sample and test runs compared to *Grin2a*$^{+/S}$ and *Grin2a*$^{+/+}$ mice (both F(2,41) >19.8;p <0.0001, and post-hoc comparisons (Tukey's HSD) p < 0.01). Discrimination in the test run: both WT (t(11) = 4.46; p < 0.002) and *Grin2a*$^{+/S}$ mice (t(15) = 3.00; p < 0.01) showed a significant preference for the novel object over the familiar object but this was not the case for the homozygous *Grin2a*$^{S/S}$ mice (t(15) = 1.33; p = 0.20; main effect of genotype F (2,41) = 2.88; p = 0.068; planned comparison *Grin2a*$^{+/+}$ vs *Grin2a*$^{S/S}$ mice p < 0.05).

f In the sample phase in the Y maze the total arm entries was significantly increased in *Grin2a*$^{S/S}$ mice compared to heterozygous and ctrl. mice (main effect of genotype; F(2,38) = 18.52; p < 0.01). In the test phase *Grin2a*$^{+/+}$ and *Grin2a*$^{+/S}$ mice showed a preference to visit the previously unexplored, novel arm of the Y-maze during whereas *Grin2a*$^{S/S}$ mice showed no novelty preference (main effect of genotype for discrimination ratio; F(2,38) = 4.53; p < 0.02; Dunnett's test for *Grin2a*$^{S/S}$ group vs. ctrls. p<0.05).

Fig. 8: *Grin2a*$^{S/S}$ mice acquire simple associative learning

a Odor learning was not affected *Grin2a*$^{S/S}$ vs. *Grin2a*$^{+/+}$ ($F[2,12]=2.061$, $p=0.17$).

b Simple visuo-tactile discrimination acquisition: Main effect of genotype $F(2,25) = 4.014$; $p = 0.0308$. Post-hoc pairwise comparisons for significant main effect of genotype (Newman Keuls – Con vs Hets $p < 0.05$, no other significant differences). Main effect of block $F(5,125) = 25.478$; $p<0.0001$. Genotype by Block $F(10,125) = 1.905$; $P = 0.0503$.

c *left,* In the Morris water maze the path length to reach the hidden platform did not decrease in *Grin2a*$^{S/S}$ mice. *Grin2a*$^{S/S}$ vs. *Grin2a*$^{+/S}$ and *Grin2a*$^{+/+}$ mice. ANOVA revealed a main effect of block ($F[5,125]=28.3$; $p<0.001$), a main effect of group ($F[2,25]=48.9$; $p<0.001$) and a groups by blocks interaction ($F[10,125]=2.8$; $p<0.005$). *Post hoc* analysis between *Grin2a*$^{S/S}$ mice and *Grin2a*$^{+/S}$ and *Grin2a*$^{+/+}$ mice ($p<0.001$) and *Grin2a*$^{+/S}$ vs. *Grin2a*$^{+/+}$ ($p>0.20$).

c *right,* In the Morris water maze probe trials *Grin2a*$^{S/S}$ mice failed to search for the platform in the goal target quadrant. ANOVA for % time in the training quadrant, Probe test 1 – $F[2,25]=3.9$; $p<0.05$; Probe test 2 – $F[2,25]=17.2$; $p<0.001$, post-hoc comparisons for *Grin2a*$^{S/S}$ mice *Grin2a*$^{+/S}$ and *Grin2a*$^{+/+}$ mice at $p<0.05$ and $p<0.001$ for probe tests 1 and 2, respectively).

d In the T-maze task for rewarded alternations *Grin2a*$^{S/S}$ mice were impaired *Grin2a*$^{S/S}$ vs. *Grin2a*$^{+/+}$ mice $F[2,25]=125.77$; $P<0.0001$ for *Grin2a*$^{S/S}$ vs. both heterozygotes and WT (pairwise Tukey's; S/S vs both other groups at $p < 0.01$).

e In the cued T-Maze: both groups were consistently scoring over 80% correct. ANOVA revealed a main effect of block ($F(18,270)=24.5$; $p<0.001$), a main effect of genotype ($F(1,15)=4.6$; $p<0.05$) but no genotype by block interaction ($F(18,270)=1.3$; $p>0.10$). Post-choice baiting during block 19 did not affect memory performance in either group, confirming that the mice were not using the smell of the milk reward to solve the task

f In the discontiguous version of the T-maze with the floor inserts task *Grin2a*$^{S/S}$ mice failed to learn *Grin2a*$^{S/S}$ vs. *Grin2a*$^{+/+}$ (ANOVA revealed a main effect of block ($F[18,270]=8.1$; $p<0.001$), a main effect of genotype ($F[1,15]=21.7$; $p<0.001$) and also a significant genotype by block interaction ($F[18,270]=3.6$; $p<0.001$). *Post hoc* analysis using simple main effects showed that there was a significant impairment in the *Grin2a*$^{S/S}$ mice from block 5 to 19 (all F's$[1,130]>4.6$; $p<0.05$).

e+f When the data from these two T-maze studies were combined into a single ANOVA there was a significant genotype by task interaction ($F[1,30]=5.1$; $p<0.05$). Subsequent analysis of simple main effects revealed a significant effect of task for the *Grin2a*$^{S/S}$ mice ($F[1,30]=13.2$; $p<0.005$) but not for WT ($F<1$; $p>0.20$).

Supplementary References

1 Nagy, A., Rossant, J., Nagy, R., Abramow-Newerly, W. & Roder, J. C. Derivation of completely cell culture-derived mice from early-passage embryonic stem cells. *Proc Natl Acad Sci U S A* **90**, 8424-8428 (1993).

2 Schwenk, F., Baron, U. & Rajewsky, K. A cre-transgenic mouse strain for the ubiquitous deletion of loxP-flanked gene segments including deletion in germ cells. *Nucleic Acids Res* **23**, 5080-5081 (1995).

3 Quinn, L. P. *et al.* LABORAS: Initial pharmacological validation of a system allowing continuous monitoring of laboratory rodent behaviour. *J Neurosci Methods* **130**, 83-92, doi:10.1016/s0165-0270(03)00227-9 (2003).

4 Hamers, F. P., Koopmans, G. C. & Joosten, E. A. CatWalk-assisted gait analysis in the assessment of spinal cord injury. *J Neurotrauma* **23**, 537-548, doi:10.1089/neu.2006.23.537 (2006).

5 Hamers, F. P., Lankhorst, A. J., van Laar, T. J., Veldhuis, W. B. & Gispen, W. H. Automated quantitative gait analysis during overground locomotion in the rat: its application to spinal cord contusion and transection injuries. *J Neurotrauma* **18**, 187-201, doi:10.1089/08977150150502613 (2001).

6 Kameda, T., Kaneuchi, Y., Sekiguchi, M. & Konno, S. I. Measurement of mechanical withdrawal thresholds and gait analysis using the CatWalk method in a nucleus pulposus-applied rodent model. *J Exp Orthop* **4**, 31, doi:10.1186/s40634-017-0105-5 (2017).

7 Kondziella, W. [a New Method for the Measurement of Muscle Relaxation in White Mice]. *Arch Int Pharmacodyn Ther* **152**, 277-284 (1964).

8 Deacon, R. M. Measuring motor coordination in mice. *J Vis Exp*, e2609, doi:10.3791/2609 (2013).

9 Sanderson, D. J. *et al.* Deletion of glutamate receptor-A (GluR-A) AMPA receptor subunits impairs one-trial spatial memory. *Behav Neurosci* **121**, 559-569 (2007).

10 von Engelhardt, J. *et al.* Contribution of hippocampal and extra-hippocampal NR2B-containing NMDA receptors to performance on spatial learning tasks. *Neuron* **60**, 846-860, doi:10.1016/j.neuron.2008.09.039 (2008).

11 Bannerman, D. M. *et al.* Dissecting spatial knowledge from spatial choice by hippocampal NMDA receptor deletion. *Nat Neurosci* **15**, 1153-1159, doi:10.1038/nn.3166 (2012).

12 Jensen, V. *et al.* A juvenile form of postsynaptic hippocampal long-term potentiation in mice deficient for the AMPA receptor subunit GluR-A. *J Physiol* **553**, 843-856, doi:10.1113/jphysiol.2003.053637 (2003).
