# Supplementary material for: Voltage-independent GluN2A-type NMDA receptor Ca2+ signaling promotes audiogenic seizures, attentional and cognitive deficits in mice
Source: Commun Biol. 2021 Jan 8;4:59. doi: 10.1038/s42003-020-01538-4 (PMC7794508; doi:10.1038/s42003-020-01538-4)
Supplement: Supplementary file 3 — Description of Additional Supplementary Files [file 42003_2020_1538_MOESM3_ESM.pdf]

Description of Additional Supplementary Files

File Name: Supplementary Data 1

Description: To Fig 1d: Sequence Grin2a+/+ 637-1.abi. Chromatogram DNA sequence analysis result of a Grin2a RT-PCR DNA fragment that was amplified from reverse transcribed whole brain RNA (cDNA) of the Grin2a+/+control mouse 637-1.

File Name: Supplementary Data 2

Description: To Fig 1d: Sequence Grin2aS/S.abi. Chromatogram DNA sequence analysis result of an Grin2a RT-PCR DNA fragment that was amplified from reverse transcribed whole brain RNA (cDNA) of a homozygous Grin2aS/S mouse.

File Name: Supplementary Data 3

Description: To Fig 1d: Sequence Grin2a+/S 637-3.abi. Chromatogram DNA sequence analysis result of an Grin2a RT-PCR DNA fragment that was amplified from reverse transcribed whole brain RNA (cDNA) of a homozygous of the heterozygous Grin2a+/S mouse 637-3.

File Name: Supplementary Data 4

Description: To Fig. 1d: Comparable Grin2a+ and Grin2aS brain mRNA levels in Grin2a+/S mice.

The sequence chromatograms of the Grin2a+/+ and Grin2a+/S derived RT-PCR sequences covering the coding region for the by gene-targeted coding (N615S) and the two non-coding mutations shows that all seven Grin2a+/S mice express the mutated and the wild-type consistently at comparable levels.

File Name: Supplementary Data 5

Description: To Fig. 1e: Protein quantification. The quantitative analysis of the signals of the Western blot shown in Supplementary Fig. 2 are presented in an Excel file.

File Name: Supplementary Data 6

Description: To Fig. 2a/b: iGluR-Currents in vitro and in vivo. (a) The NMDA and AMPA and (b) the excitability are given in an Excel file.

File Name: Supplementary Data 7

Description: To Fig. 3a/c: Body weight, Nesting, Burrowing and running wheel. (a) the body weight, the score in the nest building the collected food in g and (c) distance in a running well are given in an Excel sheet.

File Name: Supplementary Data 8

Description: To Fig. 5b: C-fos quantification. C-fos positive cells in the different brain areas of the brain slices are given together with the analysis in an Excel file.

File Name: Supplementary Data 9

Description: To Fig. 6b right: Modulation index. Calculation of the MI values for the three genotypes was calculated in an Excel sheet.

File Name: Supplementary Data 10

Description: To Fig. 7a: Locomotor activity. Table of the light beam brakes of each animal during the recording session is given in an Excel file.

File Name: Supplementary Data 11

Description: To Fig. 7b-f: Simple Associations and CAR test. (b) The activity of the animals during object exploration, (c) the number of falls in the CAR test, (d) the social interactions in the three-chamber test, (e) the object interactions in the object recognition task and (f) the number of arm entries in the novel arm Y-maze test are given in 5 Excel sheets.

File Name: Supplementary Data 12

Description: To Fig. 8a-f: Learning and Memory. The correct choice in (a) the odor recognition task and (b) in the cue discrimination T-maze task, (c) the path length to reach the platform in the Morris water maze together with the time in the different quadrants after removal of the platform T-maze, (d) the correct choices in the rewarded T-maze alternation task, (e) the contiguous and (f) the discontiguous task are given in an Excel file.

File Name: Supplementary Data 13

Description: To SFig. 3c: LTP Data. The fEPSP slopes before and after tetanic stimulation and with and without CP101,606 are given in an Excel file.

File Name: Supplementary Data 14

Description: To SFig4d: LABORAS. The automatically recorded distance and the number of detected climbing, rearing, grooming and eating events are given in an Excel file.

File Name: Supplementary Data 15

Description: To-SFig. 4e-g: The recorded motor behavior from a cohort 9 control and 11 homozygous mutants are given in an Excel file.

File Name: Supplementary Data 15

Description: To SFig. 7e: Spontaneous alternations. The number of correct spontaneous alternations of the mice is given in a n Excel sheet.

File Name: Video 1

Description: Clasping Reflex: Grin2aS/S mice show paw and limb-clasping reflex already at postnatal day 16 when picked up by the tail.

File Name: Video 2

Description: Audiogenic seizures: During exposure to a 11 kHz sound wave frequency tone all homozygous Grin2aS/S and some heterozygous Grin2a+/S mice develop epileptic seizures with a tonic phase and respiratory arrest.

File Name: Video 3

Description: Hippocampal slice preparation: From the dissected brain, after partial removal of the cerebellum and of the olfactory bulb, a scalpel and spoon were used to dissect out the area of the dorsal hippocampus for the following vibratome slicing of 400 um transverse slices.
