# Supplementary material for: Voltage-independent GluN2A-type NMDA receptor Ca2+ signaling promotes audiogenic seizures, attentional and cognitive deficits in mice
Source: Commun Biol. 2021 Jan 8;4:59. doi: 10.1038/s42003-020-01538-4 (PMC7794508; doi:10.1038/s42003-020-01538-4)
Supplement: Supplementary file 7 — Supplementary Data 4 [file 42003_2020_1538_MOESM7_ESM.pdf]

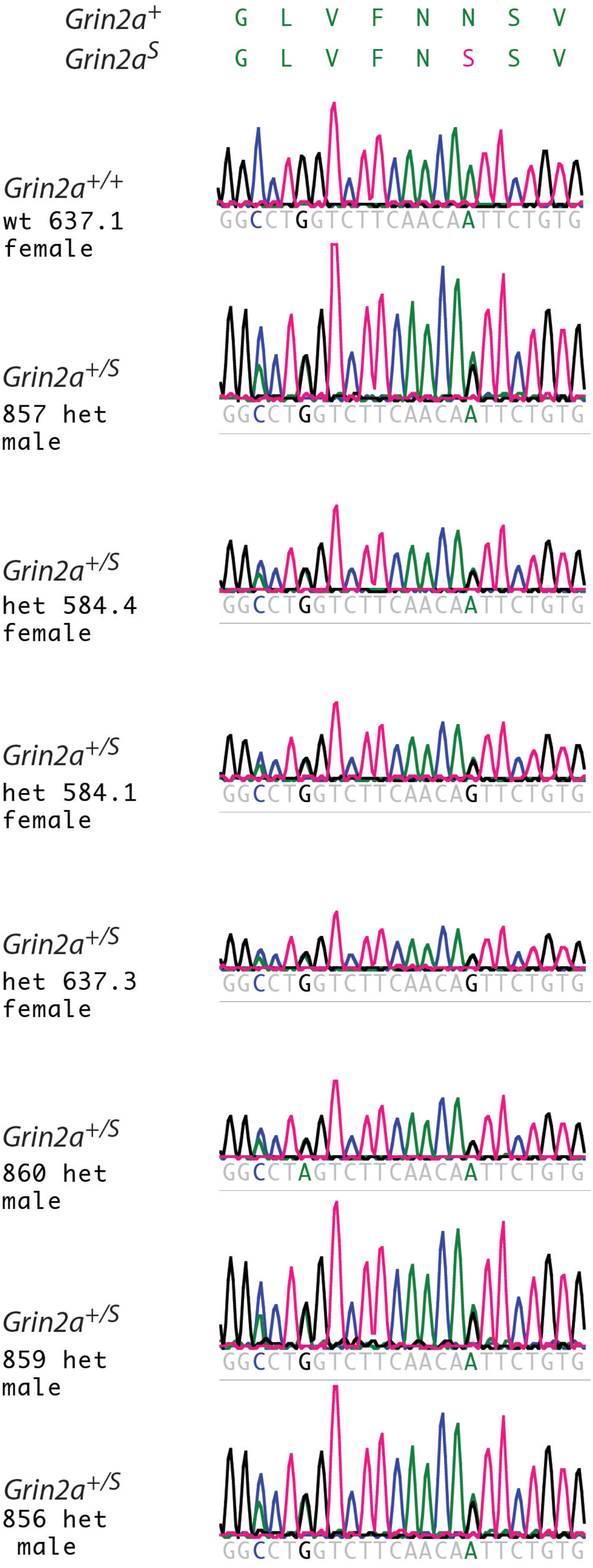
Grin2a+
G L V F N N S V
Grin2aS
G L V F N S S V
Grin2a+/+
wt 637.1
female
GGCCTGGTCTTCAACAATTCTGTG
Grin2a+/S
857 het
male
GGCCTGGTCTTCAACAATTCTGTG
Grin2a+/S
het 584.4
female
GGCCTGGTCTTCAACAATTCTGTG
Grin2a+/S
het 584.1
female
GGCCTGGTCTTCAACAGTTCTGTG
Grin2a+/S
het 637.3
female
GGCCTGGTCTTCAACAGTTCTGTG
Grin2a+/S
860 het
male
GGCCTAGTCTTCAACAATTCTGTG
Grin2a+/S
859 het
male
GGCCTGGTCTTCAACAATTCTGTG
Grin2a+/S
856 het
male
GGCCTGGTCTTCAACAATTCTGTG
